# Are neighbourhood social capital and availability of sports facilities related to sports participation among Dutch adolescents?

**DOI:** 10.1186/1479-5868-9-90

**Published:** 2012-07-31

**Authors:** Richard G Prins, Sigrid M Mohnen, Frank J van Lenthe, Johannes Brug, Anke Oenema

**Affiliations:** 1 Department of Public Health, Erasmus University Medical Center, P.O. Box 2040, 3000 CA, Rotterdam, The Netherlands; 2 National Institute for Public Health and the Environment, Centre for Prevention and Health Services Research, PO Box 1, 3720 BA, Bilthoven, The Netherlands; 3 EMGO Institute for Health and Care Research and the Department of Epidemiology & Biostatistics, VU University Medical Center, Amsterdam, the Netherlands; 4 Department of Health Promotion, Maastricht University, Maastricht, the Netherlands

**Keywords:** Sport, Adolescent, Neighbourhood social capital, Social environment, Physical environment, Availability, Parks, Interaction

## Abstract

**Background:**

The aim of this study is to explore whether availability of sports facilities, parks, and neighbourhood social capital (NSC) and their interaction are associated with leisure time sports participation among Dutch adolescents.

**Methods:**

Cross-sectional analyses were conducted on complete data from the last wave of the YouRAction evaluation trial. Adolescents (n = 852) completed a questionnaire asking for sports participation, perceived NSC and demographics. Ecometric methods were used to aggregate perceived NSC to zip code level. Availability of sports facilities and parks was assessed by means of geographic information systems within the zip-code area and within a 1600 meter buffer. Multilevel logistic regression analyses, with neighborhood and individual as levels, were conducted to examine associations between physical and social environmental factors and leisure time sports participation. Simple slopes analysis was conducted to decompose interaction effects.

**Results:**

NSC was significantly associated with sports participation (OR: 3.51 (95%CI: 1.18;10.41)) after adjustment for potential confounders. Availability of sports facilities and availability of parks were not associated with sports participation. A significant interaction between NSC and density of parks within the neighbourhood area (OR: 1.22 (90%CI: 1.01;1.34)) was found. Decomposition of the interaction term showed that adolescents were most likely to engage in leisure time sports when both availability of parks and NSC were highest.

**Conclusions:**

The results of this study indicate that leisure time sports participation is associated with levels of NSC, but not with availability of parks or sports facilities. In addition, NSC and availability of parks in the zip code area interacted in such a way that leisure time sports participation is most likely among adolescents living in zip code areas with higher levels of NSC, and higher availability of parks. Hence, availability of parks appears only to be important for leisure time sports participation when NSC is high.

## Introduction

Sports participation among adolescents is a public health priority [1,2] and increases the likelihood of being physically active in adulthood [3]. Despite the fact that levels of sports participation are relatively high among Western adolescents, a steep decrease during adolescence has repeatedly been reported [4-9]. For example, in the Netherlands it was found that 74% of the adolescents engaged in sports in the first year of secondary education, but that this dropped to 48% two years later [4]. In order to promote sports participation, deeper understanding of the factors that are associated with sports participation among adolescents is needed. In this respect, environmental factors, such as the availability of sports facilities or living in a supportive social environment, are of particular interest, as environmental factors may have an influence on the behaviour of large groups of people. Recently, there has been a call for studies to simultaneously study the social and the physical environment in order to find their independent relation and potential synergy in facilitating physical activity (PA) [10-13]. Therefore this study aims to study the social (i.e. Neighborhood Social Capital) and physical environment (i.e. availability of parks and sports facilities) simultaneously in relation to sports participation among Dutch adolescents.

For this purpose, the concept of *behaviour setting,* defined as “those social and physical situations in which behaviours take place, by promoting and sometimes demanding certain actions and by discouraging or prohibiting others” [14], is important. This implies that both social and physical environmental factors may influence PA, as also conceptualized in socio-ecological models [15,16]. For example, the EnRG framework postulates that both the physical and social environment may influence PA behaviour [15]. In addition, a model suggested by Franzini et al. elaborates more on the interplay between social and physical environmental factors and suggests that the social environment can moderate the relation between factors in the physical environment and outdoor PA [16]. In this view it is very well possible that, when facilities to be active are available, they will be used more often if the social environment is supportive instead of unsupportive.

Thus far, most empirical studies have focused on physical environmental factors, but these studies have not yet been able to nail down which physical environmental factors are directly associated with PA. Some studies have found positive associations between the objectively assessed availability of facilities to be active and PA behaviour [17-19], whilst others did not find this association [4,20,21]. It may be that the physical environment facilitates behaviour to a certain extent, but is not sufficient to motivate or enable most people sufficiently to actually engage in PA [22]; other environmental factors, such as the social environment factors may be of additional importance for PA [12,13].

Social capital is such a social environmental factor that may have an influence on sports participation. The origin of social capital states that “social networks have value” [23] and that social capital is a resource that “inheres in the structure of relations between actors and among actors.” [24]. In the present study, social capital is defined as the resources (e.g. norms, trust) that are available to all members of a community (in our case a neighbourhood) [25]. Crucial to social capital on the community level is having common norms, behavioural reciprocity and mutual trust [23]. Neighbourhood social capital (NSC) is thought to affect health related outcomes, such as leisure time sports participation, via various pathways. For instance, neighbours that trust one another are more likely to provide help and support when needed [26]; for instance by bringing each others children to sports clubs, or looking after the children when playing in parks. In addition, NSC may aid the transfer of information on healthy behaviour and maintenance of healthy norms and provision of social support [26-28]. Because neighbours live close to each other, it is likely that neighbours observe and learn from each other’s behaviour [29,30], especially when there is a strong social connection between neighbours. So, NSC may help to transfer norms, improve access to social support and provide positive role models for of behaviours such as sports participation. These norms may be healthy or unhealthy and may cause healthy or unhealthy behaviour respectively. However, the empirical evidence to date shows that the associations between measures of social capital and PA among children and adolescents are positive. Indeed, various studies confirmed that higher levels of perceived social capital were associated with higher levels of PA among children [31] and adolescents [10,11], even after being adjusted for physical environmental factors [10,32].

It is plausible that NSC and physical environmental factors can have a simultaneous or synergistic effect on PA and there are two plausible mechanisms. Firstly, it has been proposed that a supportive social environment may help to overcome an unsupportive physical environment [16]. Hence, individuals may be physically active even when some physical environmental variables are not favourable. Secondly, social capital may “add value” to the physical environment. For instance, when more people are physically active in their neighbourhood, parks and sport facilities may be more attractive places to go to. As formal amenities such as sports facilities may require memberships and informal amenities such as park do not require this, it is likely that the proposed interactions are more likely regarding informal amenities. There are only few studies that have examined these interaction mechanisms. Broyles et al. [33] have found that parks with higher levels of social capital are visited more often and the total volume of energy expenditure was also significantly higher in those parks [33]. Seaman and co-workers concluded that in order to promote access to green space in urban communities there is an interaction between physical availability of green space and urban community contexts [34].

The present study aims to explore the direct and adjusted associations of an adolescent specific measure of NSC (social environment), availability of sports facilities and parks (physical environment) with leisure time (LT) sports participation. In addition interaction of NSC on the physical environment- LT sports participation association will be explored.

We hypothesize that 1) NSC is positively and significantly associated with LT sports participation, 2) the availability of sports facilities and parks are positively and significantly associated with LT sports participation, 3) NSC and availability of sports facilities and parks interact in such a way that availability of parks and sports facilities are stronger related with LT sports participation when NSC is higher and that this effect will be more pronounced in freely available facilities such as parks.

## Methods

### Study design

The data used in this study are derived from the final post measurement of the YouRAction study (2009–2010, Rotterdam and surroundings, the Netherlands), because information on NSC was only collected in this measurement. YouRAction was a three-armed cluster randomized trial in which two versions of a computer tailored PA promotion intervention were evaluated against a generic information control group. In the first and second arm of the trial adolescents received a computer-tailored advice to promote their PA levels. In the third arm, adolescents (12–13 years) received generic information about PA and diet. The interventions are extensively described elsewhere [35]. In the trial, measurements were conducted at baseline, one month and six months post intervention (last measurement). School classes were randomly assigned to one of the study arms using block randomization. The evaluation study showed that the interventions were not effective in promoting moderate-to-vigorous PA among adolescents [36] and additional analyses showed that there were no changes in sports participation between baseline and final post intervention measurement among all students who participated in the study.

The Medical Ethics committee of the Erasmus Medical Center issued a “declaration of no objection” for the YouRAction study.

### Sampling and procedure

Schools were informed about the study and contacted to assess their willingness to participate in the trial. In participating schools (n = 12) between 1 and 12 classes (1240 students) were selected for participation in the study. All students in a class participated in the study unless they or their parents rejected to participate.

Of the 1240 adolescents who were invited to participate in the YouRAction trial, 27 (2.2%) declined to participate. In the final follow-up measurement, in total 1129 adolescents participated. Participants completed self-administered questionnaires on PA, cognitive determinants of PA, perceived environmental determinants of PA and demographics during a school hour in the presence of a research assistant and a teacher.

Adolescents with complete data on the variables of interest and living in neighbourhoods in which at least 5 respondents lived were eligible for analyses (to be able to generate a reliable aggregated value for NSC). In total 852 adolescents met these criteria.

### Measures

#### LT sports participation

Sports participation was assessed using the sports participation questions from an adapted version of the Activity QUestionnaire for Adolescents & Adults (AQUAA) [37]. The AQUAA showed moderate test-retest reproducibility, with an intra-class correlation of 0.59 for vigorous activities [37].

Adolescents could write down a maximum of 3 sports in which they had participated during the previous week, and indicate on how many days of the week (0–7 days) they had participated in each sport. Moreover, they could indicate the context in which this took place: school, neighbourhood, sports club and at home.

If sports only took place at school, the sports frequency for that particular sport was set to 0 (i.e. not participating in sports), because this does not add to LT sports participation. A dichotomized variable was created to indicate whether an adolescent participated in LT sports at least once a week (1) or not (0).

#### Neighbourhood social capital

To create an adolescent specific measure of NSC, adolescents filled in two items in the questionnaire on social capital: 1) “the people in my neighbourhood get along with each other well”, and 2) “I live in a close-knit neighbourhood with a lot of solidarity”. Response categories ranged from ‘totally disagree’ (1) to ‘totally agree’ (5) (Cronbach’s Alpha: 0.87). A measure for NSC was constructed by aggregating the individual responses from the questionnaire to the neighbourhood (defined as 4 digit zip code) level. On average 15.2 adolescents per neighbourhood answered the two NSC questions.

An ecometrics approach was used for creating the aggregated measure of social capital (for extensive information see [38-40]). In this approach, the two items measuring social capital were the dependent variables (i.e. a long dataset was created and a dummy variable indicates the item number). A linear three-level multilevel model (neighbourhoods, individuals, items), that accounts for the nesting of social capital items within individuals and neighbourhoods, was used. The model was adjusted for six individual characteristics that may influence the perception of NSC: gender, ethnicity, age, education, type of housing the adolescent lives in and years living in the current home. The residuals from this analysis, i.e. the part that cannot be attributed to individual response patterns, constitutes the NSC variable. Positive values indicate higher than average levels of NSC. The reliability of the ecometric scales depend on the variance at the three levels, i.e., items nested within respondents, and respondents nested within neighbourhoods [41]. In our sample we found a reliability of the NSC variable (based on Hox, 2002 [41]) which was acceptable at 0.57 (see additional file 1 for more details on calculation). The construction of the NSC variable was done in MLwiN 2.02.

#### Availability of sports facilities and parks

Geographic information system (GIS) data on the availability of sports facilities and parks were retrieved from municipal databases. Addresses of adolescents’ homes were geocoded by using the centroid of the six-digit zip codes of their home address.

Two measures of availability of sports facilities and parks were constructed. The first variable represents the number of sports facilities and parks within 1600 meters crow-fly buffers from the participants’ home addresses. The chosen spatial scale is in line with previous research [4] and based on a study by Colabianchi et al. [42], in which it was found that adolescents are willing to travel about 15 minutes. Given that cycling is a common mode of transport in the Netherlands, 1600 meters can be easily reached by the adolescents. However, although crow-fly buffers are commonly used in the study of the physical environment with behaviour, this variable does not match with the geographic boundaries (zip code) of the NSC variable. Therefore, a second variable was created which indicates the availability of sports facilities and parks within each 4 digit zip code area. This area is smaller than the area of a 1600 meter buffer, with a mean area of 221 (SD: 340) hectares. A standardized variable for availability in the zip code area was calculated by dividing the number of facilities by the area of the zipcode; i.e. a density measure is created. The neighbourhood scale of this variable matches with the scale used for constructing the NSC variable.

#### Covariates

Ethnicity was based on questionnaire information on country of birth of the adolescent and both parents, according to the standard definition of Statistics Netherlands [43]. An adolescent was considered to be of Western descent if he or she and both parents were born in the Netherlands, another European country, Oceania, North America, Indonesia or Japan. If the adolescent or one of the parents was born in another country, he/she was considered to be of non-Western descent.

Adolescents could indicate which level of education they attended in the questionnaire, which was categorized into higher level education (preparatory education for university) and lower level education (vocational education).

Adjustment for neighbourhood wealth is useful to mitigate area-level confounding by unmeasured factors such as neighbourhood crime and traffic safety [44]. Moreover, it is considered a convenient adjustment strategy, which entails minimal risk of over-adjustment. Therefore neighbourhood wealth was considered to be a potential area-level confounder. Neighbourhood wealth was retrieved from the WoON’09 database which is managed by the Dutch ministry of the Interior and Kingdom Relations. In this database 40.000 households are sampled and incomes per 4-digit zip code were averaged to create an overall variable of neighbourhood wealth. In addition, urbanity was considered to be an area-level confounder. Information about urbanity, measured on a zip code level was retrieved from Statistics Netherlands. The urbanity index ranges from 1 to 5, with 1 being the most rural neighbourhoods and 5 being the most urban neighbourhoods.

### Analyses

Descriptive statistics were used to describe the study population. In an empty model (i.e. model 0) we found that LT sports participation clusters within neighbourhoods (Median Odds Ratio: 1.69, 95%CI: 1.42;2.20). Therefore, multilevel regression models were used in this study, with neighbourhood and individual as levels.

Univariate two-level random intercept multilevel logistic regression (with neighbourhood and individual as levels) analyses were conducted to assess associations of demographics, NSC, availability and density of parks and sports facilities, with LT sports participation.

In order to study the association of NSC and availability/density of parks and sports facilities with LT sports participation, multiple multilevel logistic regression analyses were conducted with neighbourhood and individual as levels. In model 1, covariates, the intervention group (to adjust for potential intervention effect) and NSC were regressed on LT sports participation. In model 2, density of sports facilities and confounders were added to the empty model. The relative influence of NSC and density of sports facilities was tested by adding NSC to model 2 (model 3). In the final model (model 4), the NSC*density of sports facilities interaction term was added. The same approach was used with availability of sports facilities measured within 1600 meters and to study the influence of park density and availability on explaining LT sports participation. A significance level of p < 0.10 was applied for the interaction terms. If the interaction term between the NSC and physical environment was statistically significant, simple slope analyses were conducted to decompose the interaction effect. In order to prevent multi-collinearity in the interaction model, the availability/density of sports facilities and parks were mean centered. The NSC measure is per definition mean centered.

For all associations (except for the interaction term) a result was considered statistically significant if the p-value was lower than 0.05 for a two-sided test. All analyses were conducted in STATA 11.0.

## Results

### Descriptives and univariate associations

The sample comprised of 852 adolescents; higher educated adolescents were more likely to be in the final sample. The mean age of adolescents in the final sample was 13.24 (SD: 0.45) years, 46.2% were girls, 42.4% attained higher levels of education and 80.4% was of Western ethnic background (Table 1). In total 54.7% participated in LT sports. On average, there were 4.00 (SD: 3.63) sports facilities within a radius of 1600 meters around a participant’s home and 1.77 (SD: 1.26) parks. Univariate analyses indicated that availability of parks and of sports facilities were not associated with LT sports participation (Table 1). NSC was positively associated with LT sports participation (OR: 3.69; 95%CI: 1.19;11.45) (Table 1). Hence, an increase of one unit of NSC is associated with a 3.7 times higher odds of engaging in LT sports.

**Table 1:** Descriptives and univariate associations of demographics and environmental correlates with LT sports participation

**Factor**	**Mean (SD)/%**	**Univariate association with LT sports participation (OR, 95% CI)**
N = 852		
Age	13.25 (0.45)	1.06 (0.77;1.46)
Gender (% girls)	46.2%	**0.64** (0.48;0.86)
Education (%high)	42.4%	1.33 (0.98;1.81)
Ethnic background (%non-Western)	19.6%	0.90 (0.62;1.30)
Number of sports facilities within 1600 m	4.00 (3.63)	0.98 (0.93;1.03)
Density of sports facilities within neighbourhood	1.45 (7.82)	0.98 (0.96;1.01)
Number of parks within 1600 m	1.77 (1.26)	1.13 (0.97;1.32)
Density of parks within neighbourhood	0.98 (1.26)	1.01 (0.98;1.05)
NSC	0.00 (5.72)	**3.69** (1.19;11.45)
% participating in LT sports	54.7%	N/A

LT = leisure time; SD = standard deviation; OR = odds ratio; CI = confidence interval; NSC = neighbourhood social capital. Bold values represent statistically significant values (p < 0.05).

### NSC, availability of sports facilities and LT sports participation

Model 1 in Table 2 and 3 show that one unit increase in NSC gives a 3.5 fold increase in the odds of LT sports participation (OR: 3.51, 95%CI: 1.18;10.41), after being adjusted for potential confounder variables. No association was found for density of sports facilities in the neighbourhood (Table 2/Model 2) and availability within 1600 meter buffers (Table 3/Model 2) with LT sports participation.

**Table 2:** Associations of neighbourhood social capital and density of sports facilities in the neighbourhood with LT sports participation (OR, 95 % confidence intervals)

**N = 852/k = 56**	**Model 1 OR (95%CI)**	**Model 2 OR (95%CI)**	**Model 3 OR (95%CI)**	**Model 4 OR (95%CI)**
*Environmental factors*				
NSC	**3.51**		**3.37**	**3.28**
	(1.18;10.41)		(1.14;9,92)	(1.10;9.75)
Density of sports facilities in neighbourhood		0.99	0.99	0.99
		(0.96;1.01)	(0.97;1.01)	(0.96;1.01)
*Interaction term*				
NSC * PhysEnv				1.08
				(0.96;1.22)
*Measures of variation of clustering*				
Area level variance	0.19	0.24	0.18	0.17
	(0.06;0.59)	(0.09;0.63)	(0.06;0.58)	(0.05;0.57)
PCV	0.36	0.21	0.41	0.42
Median Odds Ratio	1.52	1.59	1.50	1.49
	(1.27;2.08)	(1.33;2.14)	(1.25;2.07)	(1.25;2.06)

All models are adjusted for intervention group, education, ethnicity, neighbourhood wealth and urbanity. N = number of adolescents, k = number of neighbourhoods, NSC = Neighbourhood Social Capital, OR = odds ratio, CI = confidence interval, PCV = Proportional change in variance relative to null-model. Bold values represent statistically significant associations (P < 0.05); italic values show significant interaction terms (P < 0.10).

**Table 3:** Associations of neighbourhood social capital and availability of sports facilities within 1600 meter Crow-fly buffers with LT sports participation (OR, 95% confidence intervals)

**N = 852/k = 56**	**Model 1 OR (95%CI)**	**Model 2 OR (95%CI)**	**Model 3 OR (95%CI)**	**Model 4OR (95%CI)**
*Environmental factors*				
NSC	**3.51**		**3.16**	2.86
	(1.18;10.41)		(1.05;9.53)	(0.92;8.90)
Availability of sports facilities within 1600 m buffers		0.97	0.98	0.99
		(0.91;1.02)	(0.93;1.03)	(0.93;1.05)
*Interaction term*				
NSC * PhysEnv				1.10
				(0.87;1.37)
*Measures of variation of clustering*				
Area level variance	0.19	0.23	0.18	0.18
	(0.06;0.59)	(0.09;0.62)	(0.06;0.58)	(0.06;0.57)
PCV	0.36	0.23	0.41	0.40
Median Odds Ratio	1.52	1.58	1.50	1.50
	(1.27;2.08)	(1.33;2.12)	(1.25;2.07)	(1.26;2.06)

All models are adjusted for intervention group, education, ethnicity, neighbourhood wealth and urbanity. N = number of adolescents, k = number of neighbourhoods, NSC = Neighbourhood Social Capital, OR = odds ratio, CI = confidence interval, PCV = Proportional change in variance relative to null-model. Bold values represent statistically significant associations (P < 0.05); italic values show significant interaction terms (P < 0.10).

In the third model, which also includes density/availability of sports facilities, NSC remained positively associated with LT sports participation for both density in the neighbourhood (OR: 3.37, 95%CI 1.14;9.92) and availability in a 1600 meter buffer size (OR:3.16, 95%CI: 1.05;9.53), while density or availability of sports facilities was not significantly associated (Table 2 and 3).

In the final model, the interaction terms, NSC*availability/density of sports facilities, were not significantly associated with LT sports participation (Table 2 and 3).

### NSC, availability of parks and LT sports participation

Density of parks in the neighbourhood (Table 4/Model 2) and availability of parks within 1600 meter buffers (Table 5/Model 2) were not significantly associated with LT sports participation.

**Table 4:** Associations of neighbourhood social capital and density of parks in the neighbourhood with LT sports participation (OR, 95% confidence intervals)

**N = 852/k = 56**	**Model 1 OR (95%CI)**	**Model 2 OR (95%CI)**	**Model 3 OR (95%CI)**	**Model 4 OR (95%CI)**
*Environmental factors*				
NSC	**3.51**		**3.52**	**3.95**
	(1.18;10.41)		(1.19;10.45)	(1.34;11.59)
Density of parks in neighbourhood		1.02	1.02	1.00
		(0.98;1.05)	(0.98;1.05)	(0.97;1.04)
*Interaction term*				
NSC * PhysEnv				*1.22*
				(0.99;1.50)
*Measures of variation of clustering*				
Area level variance	0.19	0.25	0.19	0.16
	(0.06;0.59)	(0.10;0.64)	(0.06;0.58)	(0.05;0.54)
PCV	0.36	0.16	0.37	0.47
Median Odds Ratio	1.52	1.62	1.52	1.47
	(1.27;2.08)	(1.35;2.15)	(1.27;2.07)	(1.23;2.02)

All models are adjusted for intervention group, education, ethnicity, neighbourhood wealth and urbanity. N = number of adolescents, k = number of neighbourhoods, NSC = Neighbourhood Social Capital, OR = odds ratio, CI = confidence interval, PCV = Proportional change in variance relative to null-model. Bold values represent statistically significant associations (P < 0.05); italic values show significant interaction terms (P < 0.10).

**Table 5:** Associations of neighbourhood social capital and availability of parks within 1600 meter Crow-fly buffers with LT sports participation (OR, 95% confidence intervals)

**N = 852/k = 56**	**Model 1 OR (95%CI)**	**Model 2 OR (95%CI)**	**Model 3 OR (95%CI)**	**Model 4 OR (95%CI)**
*Environmental factors*				
NSC	**3.51**		**3.45**	**3.18**
	(1.18;10.41)		(1.16;10.29)	(1.09;9.29)
Availability of parks in 1600 meter buffers		1.07	1.05	1.06
		(0.88;1.29)	(0.88;1.26)	(0.88;1.26)
*Interaction term*				
NSC * PhysEnv				1.64
				(0.66;4.05)
*Measures of variation of clustering*				
Area level variance	0.19	0.26	0.19	0.14
	(0.06;0.59)	(0.10;0.65)	(0.06;0.59)	(0.04;0.54)
PCV	0.36	0.15	0.36	0.52
Median Odds Ratio	1.52	1.62	1.52	1.44
	(1.27;2.08)	(1.35;2.15)	(1.27;2.08)	(1.21;2.02)

All models are adjusted for intervention group, education, ethnicity, neighbourhood wealth and urbanity. N = number of adolescents, k = number of neighbourhoods, NSC = Neighbourhood Social Capital, OR = odds ratio, CI = confidence interval, PCV = Proportional change in variance relative to null-model. Bold values represent statistically significant associations (P < 0.05); italic values show significant interaction terms (P < 0.10).

When adjusted for density in the neighbourhood (OR: 3.52, 95%CI: 1.19;10.45) or availability in a 1600 meter buffer size (OR: 3.45, 95%CI: 1.16;10.29), NSC was still significantly associated with LT sports participation (Table 4 and 5).

**Figure 1:**
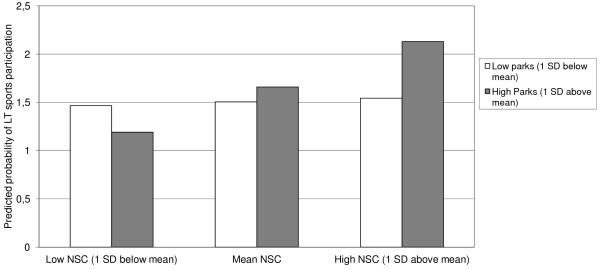
Visual presentation interaction intention at density of parks * neighbourhood social capital on LT sports participation. NSC = Neighbourhood social capital, SD = standard deviation.

In the final model, the NSC*availability of parks at 1600 meter buffer sizes variable was not significantly associated with LT sports participation (Table 4). However, density of parks within the neighbourhood did interact with NSC (OR: 1.22; 95%CI: 0.99;1.50, Table 5). To better interpret this interaction term, simple slope analyses were conducted. In this figure the probability of participating in sports is plotted for two levels of density of parks (low and high) and three levels of NSC (low NSC, 1 SD below mean; mean NSC; high NSC, 1 SD above mean). These simple slope analyses show that when both availability of NSC and density of parks are highest, the probability of participating in LT sports was highest (Figure 1).

## Discussion

In this study we found that NSC was significantly associated with LT sports participation, whereas the objectively observed availability and density of parks or sports facilities was not. The association of NSC with LT sports participation remained significant when adjusted for availability/density of sports facilities or parks. In addition we found that when NSC was high, availability of parks was associated with LT sports participation, which was in line with our hypotheses.

It is often argued that NSC has positive influences on health behaviour. However, it is theoretically also possible that negative norms that exist in a neighborhood may be “disseminated” through the community with detrimental influences on behaviour. Interesting is that higher levels of social capital may also unintentionally lead to negative influences on physical activity. In a study by Altschuler et al. [45] it was found that in one neighbourhood successfully lobbied in preventing installation of street lighting – so the natural scenery of the neighbourhood was saved. This lead to a situation which is less attractive for pedestrians and hence, walking. However, in this study, NSC was consistently positively associated with LT sports participation. This finding fits with our hypotheses, various theoretical models [15,16] that assume this association and with the results of previous research that have used a measure of perceived NSC (i.e. not aggregated to the neighbourhood level) [10,11]. The present study extends on the existing evidence, as we used an aggregated measure of NSC.

With regard to the physical environment, we did not find an association of availability and density of sports facilities or parks with LT sports participation in both areas that we studied. These results add to the current, equivocal, literature on the associations of the physical environment with adolescent PA [46]. It may be that these inconclusive findings are due to various conceptualizations of the neighbourhood. Therefore various authors have called upon reporting results in various environmental scales (i.e. buffer sizes) [47,48]. We have conceptualized the physical environment in two scales; one that is evidence based (1600 meter buffer size) and one that matches the NSC measure (neighbourhood level), but found no differences in associations between these two conceptualizations. It may be that adolescents are active in other places than the neighbourhood in which they live. It is very well possible that the environment around their school, or environments where their friends live, is more important for some adolescents. For instance, Jones et al. found that MVPA takes place outside commonly used buffers [49]. A mismatch in the environment where PA facilities are assessed and the environment where PA is actually performed may have also contributed to lack of associations found. Future research should consider using advanced technology, such as GPS with integrated accelerometry in determining the environment where adolescents are active and to determine associations between the physical environments and PA. Some preliminary research indicates that using these advances in GPS technology is feasible and promising [49-53]. However, it may also be that the physical environment in itself is not enough to promote PA, but may act more as a barrier or facilitator of PA behaviour [22]. In this study we may have found some evidence that points in that direction.

We showed that density of parks at the neighbourhood level interacted with NSC in such a way that when both NSC and density of parks were high, adolescents were most likely to participate in LT sports. Thus, indeed, density of parks can be important for sports participation, but only in combination with high levels of NSC. Our results that the combination of physical and social environmental factors results in the highest likelihood for sports participation, are in line with findings of a systematic review of qualitative studies by McCormack et al. They found that the presence of a park gives important opportunities to engage in PA, but other features of that park such as the social environment may promote park usage [54]. Seaman et al. also found in a qualitative study that for promoting park use not only the presence of a park, but also factors like social cohesion are of importance [34]. Finally Broyles et al., who found that in parks with more “Park level social capital” the overall intensity of activities performed in that park were higher [33]. However, it should be noted that the social capital on a park level is not equal to NSC as we have conceptualized it.

For promoting a healthy lifestyle (e.g. sports participation) on a population level, it is important to identify important and changeable determinants of that lifestyle [55]. We have identified NSC as a potentially important factor associated with LT sports participation, especially when density of parks is high. If our results can be replicated by others, NSC may be an important factor in relation to sports promotion. In addition, to be suitable to incorporate in interventions and policy to promote LT sports participation, it is also important to know how NSC can be promoted. Wood et al. found that various factors, such as availability of facilities like nearby shops, are positively associated with higher levels of NSC [56]. This may give valuable target points for developing interventions and policies that aim to promote NSC. In addition, various studies suggest that PA can successfully be promoted by building NSC by cooperating closely with citizens in developing policies and strategies [57,58].

Although the aim of this study was not to fully explain neighbourhood variance in LT sports participation, even in the most extended models there was unexplained neighbourhood variance. This indicates that other factors may be of interest that may explain neighbourhood variation in LT sports participation; for instance environmental factors such as presence of sidewalks and bicycle lanes (increasing opportunities to access parks or sports facilities), quality of these facilities, crime or aesthetics. Studies that are aimed at explaining neighbourhood differences in LT sports participation should thus, additionally, incorporate other factors than we did.

There is some criticism on the concept of social capital in that it is a fuzzy concept. According to Kawachi et al. this may have to do with two conceptualizations of social capital. On the one side there is conceptualization that is more individual level social capital (e.g. networks) and on the other side social capital may be conceptualized as resources available to the community [25]. We used the latter definition and therefore aggregated data to the neighbourhood level. To aggregate individual data to a neighbourhood level, it is most common to calculate the average of the items measured at the individual level per neighbourhood [59]. However, this aggregation procedure has several drawbacks. First, variables measuring social capital are based on individual perception, and it is likely that this perception is influenced by the characteristics of the respondent (e.g. time living in a neighbourhood, cultural values). Second, since the number of respondents differs per neighbourhood, the reliability of the aggregated social capital variable also differs between the neighbourhoods. Finally, the items that measure social capital are not independent of each other but nested within respondents; that is, answers on one item are likely to be associated with answers on the other item. In summary, an approach that accounts for individual differences in response to certain items, for differences in numbers of respondents on which the estimation is based, and for dependency among the items that measure social capital is needed. The ecometrics methodology is an approach that does this. And we have applied this in our study, which can be regarded as a major strength in this study.

Another strength of this study is that the behaviour (LT sports participation) studied is relevant for its context (the neighbourhood environment), because it is less likely that the home neighbourhood affects inside school sports participation than LT sports participation. In contrast to other studies, the neighbourhood perception of adolescents instead of adults was used to estimate NSC, which is another strength of this study. However, as this is the first study to use adolescent perceptions of NSC, our results need to be replicated to draw more definitive conclusions. Future studies may, given the promising results of our study also wish to further explore the construction of a more child-specific measure of NSC, as the measure that we used was derived from an instrument that was originally developed for adults. Our study has also some limitations. Firstly, the current study relies on self-reported measures of PA. Objective measures, such as accelerometers, may give more valid data on minutes spend in PA. However, accelerometers do not measure important information on the type of activity (e.g. cycling, walking, sports), which is relevant to study the influence of environmental factors on behaviour, as different factors in the environment, such as availability of facilities are likely to be associated with different types of PA behaviour specific (e.g. sports facilities may be of importance for sports, but not for walking to school) [60]. Secondly, there may be self-selection bias in our sample, as suggested by Boone-Heinonen et al. [61]; physically active families may select supportive neighbourhoods for their PA to reside. This may have altered the associations found in this study. However, a study among Dutch adults did not find evidence for self-selection bias in the Netherlands [62] and parents are most likely to choose the residential neighbourhood and not the adolescents. Therefore, we expect that self-selection bias was not a major cause of bias in this study. Another limitation is the cross-sectional design and no conclusions on causality can be drawn. To draw conclusions on causal relationships future research, using longitudinal and experimental designs need to be carried out. Moreover, in replicating our findings these studies should be specifically designed to study neighbourhood influences on sports participation; by sampling multiple neighbourhoods with maximal neighbourhood variation and small clustered samples of individuals in each neighbourhood. In addition studies may also profit from inducing changes in the environmental factors to study the effects of changing physical and social environmental factors in their relation to sports participation. Finally, the data were retrieved from the YouRAction trial, which aimed to promote MVPA among adolescents. In the evaluation study, an effect of the intervention could not be established. Furthermore, we have checked whether the interventions were associated with the outcome of interest in this study and seen that there was no effect of the interventions and have adjusted for intervention group. Therefore, it is not likely that the interventions have affected the results of this study.

To conclude, in this study we found evidence for NSC as a potentially important and robust correlate of LT sports participation among adolescents. We did not find a direct association between availability of sports facilities or parks with LT sports participation among adolescents. The interaction between density of parks in the neighbourhood and NSC showed that when NSC was high, presence of parks was stronger associated with LT sports participations. In sum, the combination of high NSC and high density of parks is associated with the highest likelihood of LT sports participation among adolescents.

## Competing interests

The authors declare that they have no competing interests.

## Authors’ contributions

RGP carried out the study and conducted the data-analysis and drafted the manuscript. AO and RGP designed and conducted the study. SM and AO participated in discussing the paper, provided methodological input, and helped to draft the manuscript. JB and FvL helped to draft the manuscript. AO and JB designed the study. All authors read and approved the final manuscript.

## Supplementary Material

Additional file 1 Calculation of Neighbourhood Social Capital. Click here for file
