# Supplementary material for: Are neighbourhood social capital and availability of sports facilities related to sports participation among Dutch adolescents?
Source: Int J Behav Nutr Phys Act. 2012 Jul 31;9:90. doi: 10.1186/1479-5868-9-90 (PMC3479015; doi:10.1186/1479-5868-9-90)
Supplement: Additional file 1 — Calculation of Neighbourhood Social Capital. [file 1479-5868-9-90-S1.doc]

**Additional file 1**

The model estimating Neighbourhood Social Capital (NSC) is as follows:

,

where, *Y_ijk_* is the response to item i of adolescent j in neighbourhood k, $\gamma_{000}$ is the grand mean of NSC, *m* is the number of social capital variables (two in total, one serves as reference), *D* are item dummies, $q$ is the number of individual level adjusters (6 in total), $X$ are the adjuster variables, *v* is the neighbourhood variance, $u$ is the individual variance, and $e$ is the item variance.

The most important parameters are the neighbourhood level residuals, *v*, which indicate the degree to which social capital of neighbourhood k differs from the grand mean,$\gamma_{000}$. These residuals constitute the NSC measure. Positive values indicate higher than average levels of NSC.

The reliability of NSC is estimated by

,

where σ^2^ is the variance on neighbourhood level, τ^2^ is the variance between individuals per neighbourhood, and ω^2^ is the variance between the items. J_k_ is the number of individuals in neighbourhood _k_. Finally, *n* is the number of items to measure NSC.

The average reliability of our ecometric-based NSC measurement is 0.57. The correlation—performed at the neighbourhood level—between an aggregated social capital measure and the ecometrics-based social capital measure is 0.80.
